# Development and validation of a cardiovascular diseases risk prediction model for Chinese males (CVDMCM)

**DOI:** 10.3389/fcvm.2022.967097

**Published:** 2022-11-18

**Authors:** Ying Shan, Yucong Zhang, Yanping Zhao, Yueqi Lu, Bangwei Chen, Liuqiao Yang, Cong Tan, Yong Bai, Yu Sang, Juehan Liu, Min Jian, Lei Ruan, Cuntai Zhang, Tao Li

**Affiliations:** ^1^BGI-Shenzhen, Shenzhen, China; ^2^Peking University Shenzhen Hospital, Clinical Research Academy, Shenzhen, China; ^3^Department of Geriatrics, Tongji Medical College, Tongji Hospital, Huazhong University of Science and Technology, Wuhan, China; ^4^School of Biology and Biological Engineering, South China University of Technology, Guangzhou, China; ^5^College of Life Sciences, University of Chinese Academy of Sciences, Beijing, China

**Keywords:** cardiovascular diseases, prediction model, Chinese males, retrospective cohort study, chronic disease prevention

## Abstract

**Background:**

Death due to cardiovascular diseases (CVD) increased significantly in China. One possible way to reduce CVD is to identify people at risk and provide targeted intervention. We aim to develop and validate a CVD risk prediction model for Chinese males (CVDMCM) to help clinicians identify those males at risk of CVD and provide targeted intervention.

**Methods:**

We conducted a retrospective cohort study of 2,331 Chinese males without CVD at baseline to develop and internally validate the CVDMCM. These participants had a baseline physical examination record (2008–2016) and at least one revisit record by September 2019. With the full cohort, we conducted three models: A model with Framingham CVD risk model predictors; a model with predictors selected by univariate cox proportional hazard model adjusted for age; and a model with predictors selected by LASSO algorithm. Among them, the optimal model, CVDMCM, was obtained based on the Akaike information criterion, the Brier's score, and Harrell's C statistic. Then, CVDMCM, the Framingham CVD risk model, and the Wu's simplified model were all validated and compared. All the validation was carried out by bootstrap resampling strategy (TRIPOD statement type 1b) with the full cohort with 1,000 repetitions.

**Results:**

CVDMCM's Harrell's C statistic was 0.769 (95% CI: 0.738–0.799), and D statistic was 4.738 (95% CI: 3.270–6.864). The results of Harrell's C statistic, D statistic and calibration plot demonstrated that CVDMCM outperformed the Framingham CVD model and Wu's simplified model for 4-year CVD risk prediction.

**Conclusions:**

We developed and internally validated CVDMCM, which predicted 4-year CVD risk for Chinese males with a better performance than Framingham CVD model and Wu's simplified model. In addition, we developed a web calculator–calCVDrisk for physicians to conveniently generate CVD risk scores and identify those males with a higher risk of CVD.

## Introduction

According to the World Health Organization, cardiovascular diseases (CVD) are the leading cause of global mortality, accounting for an estimated 17.9 million deaths (31% of all deaths) each year worldwide with an estimated 523 million prevalent CVD cases in 2019 (1). CVD is also the leading cause of death in China, accounting for 45.9% and 43.6% of all deaths in rural and urban China respectively in 2019 (2). This is more alarming given the proportion of CVD death among all death causes in China used to be 12.8% in 1957 and 35.8% in 1990 (3). China also has the highest CVD burdens internationally, with an estimated 330 million people living with CVD in 2019 (2, 4).

To reduce the CVD burden, one possible solution is to identify those at higher risk of CVD and offer them appropriate advice for a healthier lifestyle. Thus, numerous prediction models have been developed globally to estimate the risk of CVD, including the Framingham (5–7), SCORE (8), ASSIGN (9), and QRISK models (10, 11). These models were constructed based on data of mainly Caucasian participants rather than Asians, thus, may not be applicable among Chinese. Wu et al. worked out a prediction model based on the USA-PRC Collaborative Study of Cardiovascular and Cardiopulmonary Epidemiology (USA-PRC study) cohort and used the China Multicenter Collaborative Study of Cardiovascular Epidemiology (MUCA) cohort for validation (12). Yang et al. constructed a prediction model for atherosclerotic CVD (China-PAR) and externally evaluated in two independent Chinese cohort (13). All these models used a 10-year prediction period, which may seem too long for those at risk to take immediate actions. An estimated risk score in a near future (e.g., within five years) might provide a more powerful warning for the early assessment and intervention for CVDs. It was reported that adherence to a healthy lifestyle could lower the CVD burden substantially in the Chinese population (14). Researches showed that males were at higher risk of CVD than females (15, 16), thus we decided to develop a CVD risk prediction model for Chinese males (CVDMCM) that could be used by clinicians easily.

In this study, we conducted a retrospective cohort study using physical examination data from Tongji Hospital in Wuhan, Hubei Province, China. With the cohort, CVDMCM was developed following the Transparent Reporting of a multivariable prediction model for Individual Prognosis or Diagnosis (TRIPOD) statement (17). CVDMCM was internally validated and compared with established models, namely the Framingham CVD risk model and Wu's simplified prediction model (12, 18).

## Methods

### Study design

We conducted a retrospective cohort study to develop CVDMCM. The participants were Chinese males from the general population going through annual physical examination at Tongji Hospital in Wuhan, Hubei Province in central China. The physical examination department of Tongji Hospital is the biggest physical examination center in Wuhan.

Inclusion and exclusion criteria: we included Chinese males aged 30–74 years old who took physical examinations at Tongji Hospital between 2008 and 2016 and had at least one revisit record by September 2019. The baseline record was defined as the first time a participant took the examination between 2008 and 2016. At baseline, participants with the following conditions were excluded: those with a congenital heart disease and ischemic stroke, other heart diseases (i.e., rheumatic heart disease), a malignant tumor, or a history of liver or kidney failure. If the participants had more than one revisit record and no outcome event occurred by September 2019, we used their last revisit date and their free-of-CVD status. If there was an event by September 2019, we used their CVD event reporting date and CVD event status.

The study was approved by the Medical Ethics Committee at Tongji Medical College, Huazhong University of Science and Technology who waived the written informed consents (TJ-IRB20191215). Participants identified with CVD would be recommended to go to see the doctor. No intervention was provided in the physical examination center.

### Potential predictors

Based on prior knowledge, we included 17 potential predictors, including lifestyle characteristics, clinical measurements, and medical history indicators collected by the trained doctors at the physical examination center of Tongji Hospital through standardized in-person interviews (19). The potential predictors included both classic ones, such as those in the Framingham CVD model, and more modern ones, such as ankle brachial index (ABI) and brachial-ankle pulse wave velocity (baPWV). Specific measuring protocols are listed in the Supplementary material. The quadratic terms of predictors, such as systolic blood pressure (SBP), diastolic blood pressure (DBP), body mass index (BMI), and glycated hemoglobin A1c (HbA1c) were considered. The interaction effects between age and the predictors were also assessed because the effects of some factors may change with age.

### Outcomes

The CVD outcomes included coronary heart disease (CHD) and ischemic stroke. CHD was mainly diagnosed according to symptoms (mainly angina) and electrocardiography or coronary angiography. Echocardiogram, exercise stress test, coronary artery calcium scan, cardiac catheterization or chest x-ray were also used in some patients to assist the diagnosis of CHD. Patients who self-reported having coronary heart disease, coronary artery bypass grafting, coronary stent implantation, percutaneous coronary intervention, or percutaneous transluminal coronary angioplasty in the follow-up visits were also considered to have CHD. Ischemic stroke was diagnosed according to symptoms and cerebral infarction and confirmed by computed tomography or magnetic resonance imaging.

### Sample size

We calculated the minimum sample size required for the prediction model according to Riley's guidance (20). By treating CVD occurrences as time-to-event outcomes, sample size calculations were provided to (S1) estimate the overall outcome proportion with precision in follow-up (S2), target a shrinkage factor of 0.9, and (S3) target small optimism of 0.05 in the apparent R^2^. Based on the three criteria, sample size in the cohort larger than the calculated minimum sample size required was considered as sufficient. The details are shown below:

We used R 4.1.3 package “pmsampsize” for criteria S1, S2, and S3 where the anticipated R^2^ value was assumed to be 0.25, according to existing CVD risk prediction models shown in Siontis et al., with up to 20 parameters (21). Siontis et al. illustrated R^2^ statistics of 12 existing CVD risk prediction models, and all the R^2^ statistics are higher than 0.25 (21). Therefore, we made a conservative choice of R^2^ of 0.20. For the convenience of clinical application, the number of parameters in the final model tends to be no more than 10. Again, to be conservative, we set the model with up to 20 parameters. The mean follow-up and the overall event rate were calculated in our study cohort. The timepoint of interest for prediction using the newly developed CVDMCM was 4 years.

### Model development

First, physical examination records which met our inclusion criteria were exported from the electronic medical record system of Tongji Hospital. We then excluded the participants who met the exclusion criteria. A retrospective cohort was established afterwards. To deal with the missing data, we applied multiple imputations to the raw data (22). The imputations were implemented with Multivariate Imputation by Chained Equations (MICE) package in R software (23) and a single imputation dataset was used. The imputation methods were predictive mean matching (PMM) for continuous data, and logistic regression (Log-Reg) for binary data. Then, we examined the baseline characteristics for the cohorts, summarized categorical data with frequencies and percentages, as well as summarized continuous data with means and standard deviations (if normally distributed) or medians and interquartile ranges (if not normally distributed).

We constructed three models. Model 1 was constructed using multivariable Cox proportional hazards regression by the predictors the same as Framingham Risk Score for Hard Coronary Heart Disease, including age, smoking status, total cholesterol, HDL, SBP, antihypertension drug usage. The predictors in Model 2 and 3 were selected by the age-adjusted univariable Cox proportional hazards regression, and the LASSO algorithm, respectively. In LASSO, the hyperparameter lambda was the one provided minimum mean cross-validated error in the 10-fold cross validation. Then the multivariable Cox proportional hazards regressions were carried out with the corresponding selected predictors to construct the model 2 and 3. In the Cox proportional hazards regression models, we assessed the proportional hazards assumption of each predictor by examining the plots of the scaled Schoenfeld residuals against time. Any non-random pattern in the plots suggested a violation of the proportional hazard's assumptions, if not fulfilled follow-up time was split accordingly.

High-risk individuals was defined as having a risk higher than the age-standardized CVD prevalence rate of Chinese males in literature (24, 25). This would provide physicians with a cutoff to inform those individuals with high CVD risk score to change their lifestyle, take more physical activities, and pay close attention to their cardiovascular health.

### Validation and model performance evaluation

All the CVDMCMs were internally evaluated in the dataset with 1,000 times bootstrap as TRIPOD statement recommended (type 1b) (26). We compared the three models in three aspects: the overall goodness of fit was assessed by AIC; the Calibration was evaluated by Brier score; and the discrimination was examined by the Harrell's C-index. In this study, AIC was calculated by *AIC* function in *stats* R package; Brier score was calculated by *brier* function in *ModelMetrics* R package; and Harrell's C-index was calculated by *cindex* function in *dynpred* R package.

The optimal model, CVDMCM was compared with the Framingham CVD risk model and the simplified model in Wu et al. (12, 18). We generated calibration plots based on the predicted and 4-year observed CVD risks. The samples were grouped into deciles according to the estimated risks. In each decile, the expected risk was calculated by taking the mean value of the estimated risks of the samples, while the observed risk was the percentage of CVD occurred at the four-year time point. Discrimination was also compared *via* calculation of Harrell's C statistic and D statistic. For the model to be acceptable, its Harrell's C statistic should be at least 0.7. If its Harrell's C statistic is over 0.9, it shows that the model has excellent predictive power (27). The D statistic is also a measure of discrimination. A higher value of D statistic indicates better discrimination (28). Harrell's C statistic and D statistic were calculated using *concordance.index* function and *D.index* function in R package *survcomp*, respectively.

All the analyses were performed using R Statistical Software, version 4.1.3. The R code is provided in Supplementary material.

## Results

### Participants characteristics

**Figure 1:**
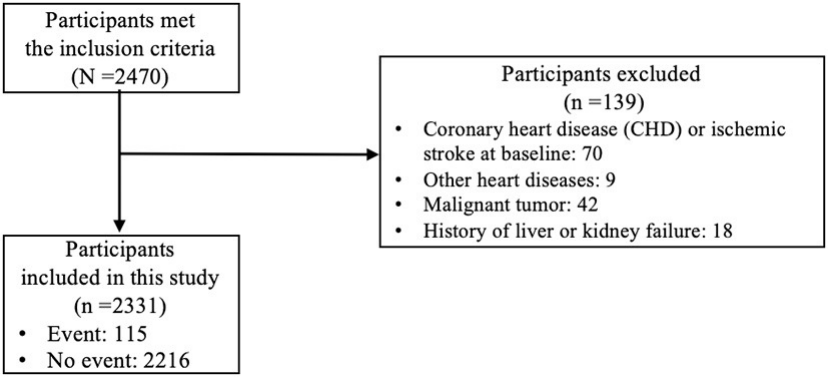
Study flow diagram.

Initially, 2,470 participants met the inclusion criteria. From those 2,470 participants, we excluded 139 participants who met the exclusion criteria, which made 2,331 participants remained in this study. The study flow diagram could be found in Figure 1. All predictors that were considered as candidates for model development were listed in the Table 1. Table 1 showed that the participants were followed for a median of 4.0 years. The median baseline age of the participants was 52 years old with the interquartile range (IQR) of 46–58 years old. The baseline age of the 130 participants who had CVD events during the follow-up was 62 (IQR: 57–66) years old, while the baseline age of the 2,201 participants without CVD events was 51 (IQR: 46–58) years old. The incidence rate of CVD per 1,000 person-years was 13.16 (95% CI: 11.00–15.63).

**Table 1:** Baseline characteristics.

**Characteristic**	**Overall**	**Non-CVD cases**	**CVD cases**	***P* value**
No. of participants, *n* (%)	2,331	2,201 (94.4%)	130 (5.6%)	
Follow-up time (years)	1,387.00 [741.00, 2,197.00]	1,393.00 [744.00, 2,197.00]	1,122.00 [635.25, 2,168.75]	0.026
Age (y)	52.00 [46.00, 58.00]	51.00 [46.00, 58.00]	62.00 [57.00, 66.00]	<0.001
BMI (kg/m^2^)	25.10 [23.50, 26.80]	25.20 [23.50, 26.90]	24.60 [23.20, 26.10]	0.006
Heavy drinking, yes, *n* (%)	478 (20.5)	471 (21.4)	7 (5.4)	<0.001
Smoking, yes, *n* (%)	774 (33.2)	739 (33.6)	35 (26.9)	0.142
ABI	1.11 [1.07, 1.17]	1.11 [1.06, 1.17]	1.12 [1.07, 1.17]	0.875
baPWV (cm/s)	1,372.00 [1,242.00, 1,536.50]	1,363.00 [1,234.00, 1,531.00]	1,485.50 [1,376.50, 1,686.12]	<0.001
eGFR (ml/min/1.73m^2^)	85.80 [76.80, 96.30]	85.90 [76.80, 96.60]	80.40 [74.90, 89.55]	<0.001
Hemoglobin A1c (mmol/mol)	38.91 [36.72, 41.09]	38.91 [36.72, 41.09]	40.00 [36.99, 43.28]	0.017
HDL (mmol/L)	1.14 [0.99, 1.31]	1.14 [0.99, 1.31]	1.15 [1.00, 1.37]	0.395
SBP (mmHg)	126.00 [117.00, 137.00]	126.00 [117.00, 136.00]	130.00 [121.00, 140.00]	0.001
SII	338.49 [252.79, 444.42]	338.49 [252.73, 443.04]	340.00 [253.70, 482.31]	0.505
Total bilirubin (μmol/L)	12.70 [10.20, 16.00]	12.80 [10.20, 16.10]	11.45 [9.45, 15.10]	0.006
Total cholesterol (mmol/L)	4.74 [4.19, 5.29]	4.75 [4.19, 5.29]	4.64 [4.02, 5.26]	0.374
Urine protein, yes, *n* (%)	100 (4.3)	91 (4.1)	9 (6.9)	0.193
Diabetes, yes, *n* (%)	257 (11.0)	237 (10.8)	20 (15.4)	0.136
Hypertension, yes, *n* (%)	638 (27.4)	595 (27.0)	43 (33.1)	0.161
Antihypertensive drug, yes, *n* (%)	79 (3.4)	72 (3.3)	7 (5.4)	0.296

IQR, interquartile range; BMI, body mass index; ABI, ankle brachial index; baPWV, brachial-ankle pulse wave velocity; eGFR, estimated glomerular filtration rate; HbA1c, glycated haemoglobinA1c; HDL-C, high-density lipoprotein cholesterol; SBP, systolic blood pressure; SII, systemic immune-inflammation index.

Continuous variables are described as median [interquartile range] and categorical variables are presented as n (percentages).

### Sample size calculation

Then, we calculated the minimum sample size required by using *pmsampsize* R package with the parameters of R^2^ value assumed to be 0.20 with up to 20 parameters. The mean follow-up of 4.23 years and the overall event rate of 0.049 were obtained in our study cohort. The timepoint of interest was 4 years. The calculated minimum sample size required was 796, which indicated that the sample size in the cohort of 2,331 was sufficient.

### Model construction, comparison and internally validation

Three models were developed and compared. In all the three models, proportional hazard assumption was satisfied. In model 2, five predictors (predictors were age, smoking status, log-transformed total bilirubin, ABI, SBP, and the interaction of age and SBP) were associated with CVD in the age-adjusted univariate Cox regressions, thus were selected. The results of age-adjusted univariable statistics were provided in Supplementary Table 1. In model 3, the weights of predictors were shrunk to 0, except for the following predictors: hyperlipidemia drug usage, age, BMI, BMI^2^, average platelet volume, log total bilirubin, ABI, interaction of age and alcohol consumptions, interaction of age and smoking, interaction of age and urine protein, interaction term of age and fasting glucose, and interaction of age and DBP.

All the CVDMCMs were internally evaluated in the whole dataset with 1,000 times bootstrap. The results were shown in Table 2. Compared to model 1 and 3, Model 2 was optimal with the smallest AIC (1,689.981, 95% CI: 1,471.859–1,965.492), BIC (1,706.728, 95% CI: 1,477.919–1,964.533) and Brier's score (0.05, 95% CI: 0.044–0.057), and the biggest Harrell's C statistic (0.769, 95% CI: 0.738–0.799). In addition, Model 2 contained less predictors than the other models, thus, more convenient for clinical usage. Thus, we selected model 2 as the final CVDMCM. The model is shown below:


$$\begin{array}{l} 4-\text{year CVD risk }\left(\text{\%}\right)\;=\;\left(1\;0.977^{\exp\left(-13.092+\text{B}\right)}\right)\\ \times \;100\text{\%},\text{in which} \end{array}$$

$$\begin{array}{l} \text{B}=\;0.305\;\times \text{ age }+\;0.364\;\times \text{ smoking }-\;0.606\;\\ \times \ln\;\left(\text{total bilirubin}\right)+\;0.093\;\times \text{ SBP}\\ 0.002\;\times \text{age }\times \text{ SBP }-\;2.528\;\times \text{ ABI} \end{array}$$

We calculated the age-standardized CVD prevalence rate of Chinese males according to Liu et al., which was 3.14 (23). An individual with an estimated risk higher than 3.14% is defined as a high-risk individual. In the cohort, 1,053 out of 2,331 individuals would be classified in the high-risk group.

**Table 2:** The comparison results of the three models.

**Predictors**	**Model 1**	**Model 2**	**Model 3**
Formula	1 – 0.975^∧^exp(0.1*age + 0.365*smoking_status −0.01*total_cholesterol −0.316*log_HDL – 0.001*SBP + 0.027*drug_hypertension – 4.953)	1 – 0.977^∧^exp(0.305*Age + 0.364*Smoking_status −0.606*log(Total_bilirubin) + 0.093*SBP – 0.002*age*SBP −2.528*ABI −13.092)	1 – 0.976^∧^exp(0.562*drug_hyperlipidemia + 0.068*age + 0.074*BMI - 0.078*average_PLT_volume −0.585*log_total_bilirubin + −2.217*ABI −0.003*BMI^2^ – 0.016*age*drinking + 0.008*age*smoking + 0.005*age*urine_protein + 0.005*age*fasting_glucose + 0.001*age*DBP – 0.258)
AIC	1,726.262 (1,508.335, 1,955.087)	1,689.981 (1,471.859, 1,965.492)	1,696.376 (1,439.917, 1,969.253)
BIC	1,713.685 (1,498.278, 1,965.748)	1,706.728 (1,477.919, 1,964.533)	1,740.198 (1,526.812, 1,976.278)
Brier's score	0.050 (0.044, 0.056)	0.05 (0.044, 0.057)	0.049 (0.042, 0.056)
Harrell's C statistic	0.763 (0.732, 0.798)	0.769 (0.738, 0.799)	0.761 (0.731, 0.788)

SBP, Systolic blood pressure; DBP, Diastolic Blood Pressure; ABI, ankle brachial index; HDL, high density lipoprotein; AIC, Akaike information criterion; BIC, Bayesian Information Criterion.

The ^∧^ symbol indicates the exponential of.

The * symbol indicates multiply.

The + symbol indicates plus.

### Compared the CVDMCM with Framingham and Wu's model

**Figure 2:**
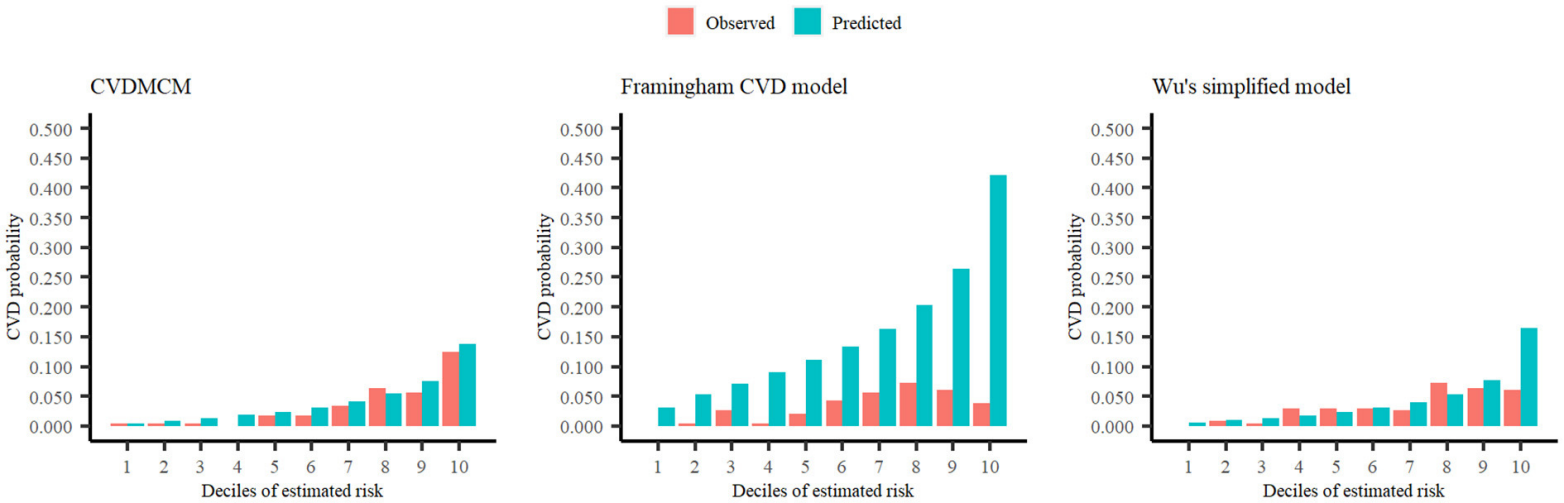
Calibration plots of CVDMCM, the Framingham CVD risk model, and Wu's simplified model for observed and predicted 4-year risks of CVD using a validation dataset.

We then compared the CVDMCM model performance with Framingham and Wu's model by bootstrap resampling the full cohort with 1,000 replicates. We first compared the predictors of CVDMCM, the Framingham CVD risk model, and Wu's model. As shown in Table 3, the predictors used in all three models included age, smoking status, and SBP. Compared with Framingham and Wu's models, CVDMCM included more modern predictors, such as ABI and Total bilirubin. We then compared the calibration of the 3 models. As shown in Figure 2, CVDMCM demonstrated higher agreement between 4-year predicted risk and observed risk, which indicated better calibration than the Framingham and Wu's simplified model. Finally, discrimination was assessed *via* Harrell's C statistic and D statistic. As shown in Table 4, CVDMCM performed better than the Framingham CVD risk model and Wu's simplified model with higher Harrell's C statistic of 0.769 (CI: 0.738, 0.799), and D statistic of 4.738 (CI: 3.270–6.864). The Harrell's C statistic of CVDMCM was significantly higher than that of Framingham CVD risk model and that of Wu's simplified model with both of the one-sided *P*-values smaller than 0.001.

**Table 3:** Predictors used in CVDMCM, the Framingham and Wu's model for males.

**Predictors**	**CVDMCM**	**Framingham**	**Wu's** **model**
Age	√	√	√
Smoking status	√	√	√
Antihypertensive medication		√	
Diabetes		√	√
BMI			√
SBP	√	√	√
ABI	√		
Total cholesterol		√	√
HDL		√	
Total bilirubin	√		

BMI, body mass index; SBP, Systolic blood pressure; ABI, ankle brachial index; HDL, high density lipoprotein.

**Table 4:** Performance of CVDMCM, the Framingham and Wu's model in the validation cohort for predicting 4-year risk of CVD.

**Validation statistics**	**CVDMCM**	**Framingham**	**Wu's model**
Harrell's C statistic	0.769 (0.738, 0.799)	0.680 (0.633, 0.727)	0.686 (0.636, 0.736)
Comparison	-	<0.001^*^	<0.001^*^
D statistic	4.738 (3.270, 6.864)	2.478 (1.731, 3.547)	2.617 (1.823, 3.757)

The brackets represent 95% confidence interval of corresponding validation statistic.

All the statistics were estimated by 1,000 iterations bootstrap resampling.

* Two-sided significant difference in Harrell's C statistic (P < 0.025) compared with the CVDMCM model.

### Model illustration

For an easier application of CVDMCM among the clinicians, we provided one example of an individual Mr. X's 4-year CVD risk. A 54-year-old Chinese man, Mr. X, who is a non-smoker with a total bilirubin of 12.30 μmol/L, ABI of 3.39, and SBP of 130mmHg, has a 4-year CVD risk of 2.39%. The calculation is shown below:


$$\begin{array}{l} 4-\text{year CVD risk }\left(\text{\%}\right)=\;\left(1\;0.977^{\exp\left(-13.092+\text{B}\right)}\right)\\ \times \;100\text{\%},\text{where} \end{array}$$

$$\begin{array}{l} \text{B }=\;0.305\;\times \text{ age }+\;0.364\times \text{smoking }-\;0.606\\ \times \text{natural}\log\left(\text{total bilirubin}\right)+\;0.093\;\times \text{ SBP}\\ \;0.0015\;\times \text{ age }\times \text{ SBP }-\;2.528\times \text{ABI}\\ =\;0.305\;\times \;54\;+\;0.364\;\times \;0\;0.606\;\times \;\log\left(12.3\right)\\ +\;0.093\;\times \;130\;0.0015\;\times \;54\;\times \;130\\ \;2.528\;\times \;3.39\\ =\;13.125 \end{array}$$

So, the 4-year CVD risk is


$$\begin{array}{l} 4-\text{year CVD risk }\left(\text{\%}\right)=\left(1\;0.977^{\exp\left(-13.092+13.125\right)}\right)\\ \times \;100\text{\%}=2.37\text{\%} \end{array}$$

Since his CVD risk is lower than 3.14%, he would be classified in the low-risk group. If someone has a CVD risk calculated higher than 3.14%, he would be classified in the high-risk group, and the physicians taking the physical examination could advise him for a healthier lifestyle. To make the CVDMCM more convenient for the physicians, we have developed an online calculator calCVDrisk (https://ctan2020.github.io/-calCVDrisk-/) for the physicians to simply enter the parameters obtained from the physical examinations, and the calculator would report the score for the physicians to assess the CVD risks.

## Discussion

Through this retrospective cohort study, we developed and validated a CVD risk prediction model CVDMCM. Unlike models with tens of predictors, CVDMCM had only five predictors (age, smoking status, total bilirubin, ABI, and SBP) and demonstrated a better performance than that of the Framingham CVD risk model and Wu's simplified model in the internal validation by bootstrap resampling the full cohort as found in Harrell's C statistic, D statistic, and calibration plot (12, 18).

There were several strengths of this study. First, we targeted a comparatively high-risk population for CVD, namely Chinese males. Given that CVD is the leading cause of death in China accounting for over 43.6% of all deaths in China, which was an increase from 12.8% in 1957 and 35.8% in 1990, immediate action is needed to change the increasing trend (3). Among the Chinese population, Chinese males were at higher risk of CVD (16). Thus, we decided to work on a model for the Chinese males in this study. Secondly, we used a 4-year prediction model. Previously, CVD prediction models were often 10-year risks, which may seem too long for those at risk to take immediate actions (8, 12, 13, 18). We hypothesized that an estimated risk score in 4 years could provide a more powerful early warning for the lifestyle change. Thirdly, the models were selected by data driven strategy according to AIC, AUC, and R^2^ indices, which was more objective. Fourth, for an easier application of our finding, we have developed an online calculator calCVDrisk for physicians to assess the CVD risk scores among those taking physical examinations.

Compared with the classic CVD models, such as the Framingham (5–7), SCORE (8), ASSIGN (9), and QRISK models (10, 11), CVDMCM was developed using Chinese males' data, which is more applicable in China. A previous study reported that CVD models should better be developed among specific populations (29). Although Wu's model is a very well designed one among the Chinese, it was published 15 years ago, thus, did not include more modern predictors such as ABI and baPWV. ABI was found as one of the five predictors in CVDMCM. ABI has become widely used in modern clinical practice and has been reported to be a sensitive measurement of CVD risk. Alves-Cabratosa et al. reported that low ABI would increase the risk of acute myocardial infarction and ischemic stroke among asymptomatic people, as well as people with diabetes and previously-diagnosed CVD (30). Velescu et al. showed that adding ABI to the Framingham CVD risk model would improve the capacity of predicting CVD events in northeastern Spain population (31). The baPWV, a parameter for the arterial stiffness assessment, has been reported as a CVD risk predictor in Japanese population (32). Therefore, we included ABI and baPWV as candidate predictors for CVDMCM in our study.

In our study, individuals in CVD group had older age, higher baPWV, lower eGFR, higher hemoglobin A1c, higher SBP, lower total bilirubin than those in Non-CVD group. Higher baPWV indicates arteriosclerosis, and higher hemoglobin A1c indicates abnormal glucose metabolism. These two indicators have been proven to be associated with higher risk of CVDs (32, 33). Lower eGFR indicates worse renal function, which may be the results of arteriosclerosis (34) or abnormal glucose metabolism (35). However, after adjusting for covariates, in the final CVDMCM model, age, smoking and SBP were included as positive predictor of CVDs, and total bilirubin and ABI were included as negative predictor. Age, smoking and SBP are well-recognized risk factor of CVDs. Interestingly, serum total bilirubin was included in the CVDMCM model but not diabetes mellitus or total cholesterol. A meta-analysis demonstrates that higher serum total bilirubin was an independent protective factor, independent of traditional risk factors, for arteriosclerotic CVDs and negatively associated with the prognosis of stroke, acute myocardial infarction, and peripheral arterial disease, but positively associated with in-hospital cardiovascular death and major adverse cardiac events (36). Similarly, total bilirubin was also a negative predictor of CVDs in our study. Bilirubin is an endogenous antioxidant, which resists for oxidative modification of low-density lipoprotein cholesterol, participates in clearing reactive oxygen species (ROS), and increases the ability of serum cholesterol dissolution (37, 38). Bilirubin can effectively block the generation of cellular ROS, and further prevent the formation of atherosclerotic plaque. Moreover, bilirubin partly inhibits the induction of complement through anti-apoptosis (39), regulates the activity of various T lymphocytes (40) and the production of proinflammatory cytokines (41).

Total cholesterol and diabetes mellitus were not included in the model. Zhu et al. indicated that aging is considered as a major risk factor for development of type 2 diabetes (42). Nunes et al. showed that age triggered increased plasma concentrations of triglycerides, cholesterol, low-density lipoproteins and lower capacity of high-density lipoproteins to remove cellular cholesterol in humans (43). Therefore, in our study, age as a potential confounder might remove the effect of total cholesterol and diabetes mellitus to the CVD endpoint when adjusting for covariates.

To better utilize CVDMCM, we developed an online calculator calCVDrisk for physicians to calculate the CVD risk of a Chinese male when taking the physical examination. We expect that CVDMCM will be promising in the application as it is a simple model with only five predictors. A previous study has shown that physicians were more willing to use simpler models in clinical practice than more complex ones when they had comparable performance (44). The estimated 4-year CVD risk will let people be alert to their cardiovascular health. A prospective cohort is under plan to use CVDMCM to detect those at higher risk of CVD to further validate the model at the physical examination department of Tongji Hospital. If the results are promising, we would advocate this application in major physical examination departments in China.

We believe that the CVDMCM could be helpful for medical doctors in the physical examination departments in China to identify those at higher risks of CVD in 4 years. For health care providers, the integration of a better CVD risk prediction model in their workflow would enable them to track the health statuses of those with high-risk scores so that doctors could provide advice for lifestyle changes and potentially help reduce CVD risks in those patients.

We admit that there are some limitations of this study. First, our model was targeting Chinese males only. As people of different ethnical background would have different risk factors, it is better to have tailor-made models for different populations. No females were included as our aim was to construct the CVD prediction model of Chinese male, given their higher risk of developing CVD (45). Second, there's no CVD caused mortality record in our data. Thus, Framingham CVD risk model and Wu's model may not be comparable. The conclusion of the better performance of CVDMCM model is based on the different end-point events. We did not compare CVDMCM to China-PAR due to lack of predictors in our physical examination data (13). Third, there is inevitably selection bias by choosing the participants taking physical examinations. They might take better care of their health than those who do not take regular physical examinations. A model to identify those at higher risk of CVD with appropriate lifestyle change advice might work with this population, as they pay more attention to their health and are more likely to follow the doctors' advice and modify their lifestyles than those who do not regularly take physical examinations. If the model works for this population, we could test it on other populations in the future. Fourth, the data obtained from electronic medical records from only one hospital may induce selection bias because it may not represent the general population of China. However, as Tongji Hospital locates in the city of Wuhan, which is considered as the geographic midpoint of China, the location might well represent the majority of Chinese cities with patients coming from all over China. The electronic medical records are from the physical examination department of Tongji Hospital, which is the biggest physical examination center in Wuhan where various groups of people choose to go to take the medical examination. Thus, the data may well represent Chinese males. Fifth, the diagnosis methods for CHD varied among different patients, some patients might undergo several kinds of examinations before the diagnosis. Although coronary angiography is a gold-standard examination for the diagnosis of CHD, some CHD patients were still diagnosed by symptoms, electrocardiography and some other examinations or tests without coronary angiography. These may bring potential bias. Sixth, the study has a bias of a loss to follow-up, which should be improved in future studies. Seventh, due to the retrospective and observational nature of our study, participants were not fully further investigated to unambiguously determine cardiovascular end points, which may bring bias. Last but not least, death information was not recorded in this study, thus, we were not able to take death as competing risks into account. However, an existing of competing risks overestimates the risks (46). This phenomenon was not presented in the calibration plot, which indicated that omitting the competing risks did not affect the prediction model much.

## Conclusion

We conducted a retrospective cohort study for developing and validating a novel CVD prediction model, CVDMCM. CVDMCM predicted 4-year CVD risk with a better performance than that of the Framingham CVD model and with fewer predictors than those involved in the Wu's simplified model. Thus, this model could be helpful in clinical practices to detect patients at higher risk of CVD, providing appropriate feedback for patients' health.

## Data availability statement

The raw data supporting the conclusions of this article will be made available by the authors, without undue reservation.

## Ethics statement

The studies involving human participants were reviewed and approved by Medical Ethics Committee at Tongji Medical College, Huazhong University of Science and Technology. The patients/participants provided their written informed consent to participate in this study.

## Author contributions

YSh, YaZ, CZ, and TL conceived the project and designed the research study. LR, YuZ, and YSa collected the data of the cohort. CT developed the website. YSh, YL, BC, and LY analyzed data. YSh, YuZ, YaZ, YL, BC, YB, and JL wrote the manuscript. All authors contributed to the article and approved the submitted version.

## Funding

This work was supported by the National Key Research and Development Program of China (Grant Number 2020YFC2008002) and Major Technology Innovation of Hubei Province (Program No. 2019ACA141).

## Conflict of interest

YSh, YaZ, YL, BC, LY, CT, YB, JL, MJ, and TL were employed by BGI-Shenzhen. The remaining authors declare that the research was conducted in the absence of any commercial or financial relationships that could be construed as a potential conflict of interest.

## Publisher's note

All claims expressed in this article are solely those of the authors and do not necessarily represent those of their affiliated organizations, or those of the publisher, the editors and the reviewers. Any product that may be evaluated in this article, or claim that may be made by its manufacturer, is not guaranteed or endorsed by the publisher.

## Author disclaimer

The contents do not reflect official views from the Hubei provincial government.
