# Supplementary material for: Development and validation of a cardiovascular diseases risk prediction model for Chinese males (CVDMCM)
Source: Front Cardiovasc Med. 2022 Nov 18;9:967097. doi: 10.3389/fcvm.2022.967097 (PMC9716142; doi:10.3389/fcvm.2022.967097)
Supplement: Supplementary file 1 [file Data_Sheet_1.docx]

***Supplementary Material***

**Online Methods**

**Measurement of clinical variables**

**Lifestyle variables**

Lifestyle variables included age, BMI, drinking, and smoking. BMI was computed as weight in kilograms divided by the square of height in meters. Drinking status was divided into two levels: 1) non-drinkers (people who never drink or who drink less than once a month, and alcohol content less than 10%); 2) drinkers (people who drink equal or more than once a month or alcohol content more than 10%). Smoking status was divided into three levels: 1) non-smokers (people who have never smoked before) or past smokers (people who used to smoke but have now quit); 2) current smokers (people who still smoke).

**Medical history**

Three medical history records, including hyperlipidemia, hypertension, and diabetes mellitus were obtained from in-person interviews. The diagnosis of hyperlipidemia was based on the history of lipid-lowering drug use or any one of the following: 1) LDL cholesterol (LDL-C) concentration of ≥4.14 mmol/L; 2) total cholesterol concentration of >6.45 mmol/L; 3) TG concentration of ≥2.26 mmol/L. The diagnosis of hypertension was based on resting blood pressure and a history of antihypertensive drug use. If the resting blood pressure was >140/90 mmHg, or with a history of antihypertensive drug use, the individual was diagnosed with hypertension. The diagnosis of diabetes mellitus was based on the history of antidiabetics drug use or any one of the following: 1) fasting plasma glucose concentration of ≥7.0 mmol/L; 2) plasma glucose concentration of ≥11.1 mmol/L 2 hours after a 75-g oral glucose load in a glucose tolerance test; 3) symptoms of high blood sugar and a casual plasma glucose concentration of ≥11.1 mmol/L; 4) HbA1c of ≥48 mmol/mol.

**Physical examination**

Blood pressure, baPWV, and ABI were measured using the Vascular Profiler BP-203RPEIII (Omron, Kyoto, Japan). The examination room was maintained at a standardized temperature of approximately 26℃. Trained technicians placed four pressure cuffs on the subjects (one on the upper part of each arm and one on each ankle). Then subjects were examined after 10 minutes of rest in the supine position. The device simultaneously recorded bilateral systolic and diastolic blood pressure, ABI, and baPWV, the latter of which was calculated as the ratio of travelled distance (which was automatically estimated from body height) divided by the transit time of the pulse wave between the brachial and posterior tibial arteries. The average of two-sided baPWV values and two-sided ABI values were recorded for analysis.

**Blood examination**

Blood examination, including routine blood tests and blood biochemical index tests, were measured using fasting venous blood samples. Routine blood tests were performed using the XN9000 (Sysmex, Kobe, Japan). Blood biochemical indices, including liver function, renal function, blood lipid profile, fasting blood glucose, HbA1c, and uric acid, were measured using the COBAS 8000 c701 (ROCHE, Basel, Switzerland).

**Urine examination**

Urinary elements were measured using the UF-1000i fully automatic urine analyzer (Sysmex, Kobe, Japan). Urinary chemistry elements were measured using the Siemens Atlas Urine Chemistry Analyzer (Siemens, Erlangen, Germany). The estimated glomerular filtration rate was calculated according to the Cockcroft–Gault formula.

**The online calculator**

We developed an online calculator for physicians to conveniently input patient data and generate CVD risk scores. The online calculator can be found on the website:

https://ctan2020.github.io/-calCVDrisk-/

**R code for model development and validation**

**library(boot)**

**library(ResourceSelection)**

**library(pROC)**

**library(survcomp)**

**library(dynpred)**

**library(ModelMetrics)**

**library(car)**

**data = read.csv("knowledge_data_one_timeV3.csv",head=T)**

**#data preparation:**

**data$time = NA**

**N = dim(data)[1]**

**for(i in 1:N){**

**data$time[i] = difftime(data$final_time_date[i],data$baseline_time[i],units = "days")**

**}**

**data$HDL = log(data$HDL)**

**colnames(data)[which(colnames(data) == "HDL")] = "log_HDL"**

**data$SII = log(data$SII)**

**colnames(data)[which(colnames(data) == "SII")] = "log_SII"**

**data$RBC_distribution_width = log(data$RBC_distribution_width)**

**colnames(data)[which(colnames(data) == "RBC_distribution_width")] = "log_RBC_distribution_width"**

**data$Fasting_blood_glucose = log(data$Fasting_blood_glucose)**

**colnames(data)[which(colnames(data) == "Fasting_blood_glucose")] = "log_Fasting_blood_glucose"**

**data$Triglyceride = log(data$Triglyceride)**

**colnames(data)[which(colnames(data) == "Triglyceride")] = "log_Triglyceride"**

**data$Total_bilirubin = log(data$Total_bilirubin)**

**colnames(data)[which(colnames(data) == "Total_bilirubin")] = "log_Total_bilirubin"**

**data$baPWV = log(data$baPWV)**

**colnames(data)[which(colnames(data) == "baPWV")] = "log_baPWV"**

**data$log_baPWV2 = data$log_baPWV^2**

**data$BMI_sq = data$BMI^2**

**data$SBP_sq = data$SBP^2**

**data$DBP_sq = data$DBP^2**

**data$Hemoglobin_A1c_sq = data$Hemoglobin_A1c^2**

**data$age_drinking = data$Age*data$Drinking**

**data$age_smoking = data$Age*data$Smoking_status**

**data$age_Urine_protein = data$Age*data$Urine_protein**

**data$age_hypertension = data$Age*data$hypertension**

**data$age_diabetes = data$Age*data$diabetes**

**data$age_hyperlipidemia = data$Age*data$hyperlipidemia**

**data$age_bmi = data$Age*data$BMI**

**data$age_bmi2 = data$Age*data$BMI_sq**

**data$age_averagePLT = data$Age*data$Average_PLT_volume**

**data$age_rbc = data$Age*data$log_RBC_distribution_width**

**data$age_sii = data$Age*data$log_SII**

**data$age_fasting_glu = data$Age*data$log_Fasting_blood_glucose**

**data$age_hemoA1c = data$Age*data$Hemoglobin_A1c**

**data$age_hemoA1c2 = data$Age*data$Hemoglobin_A1c_sq**

**data$age_hdl = data$Age*data$log_HDL**

**data$age_ldl = data$Age*data$LDL**

**data$age_Tcholesterol = data$Age*data$Total_cholesterol**

**data$age_Triglyceride = data$Age*data$log_Triglyceride**

**data$age_serum_uric_acid = data$Age*data$Serum_uric_acid**

**data$age_egfr = data$Age*data$eGFR**

**data$age_Tbilirubin = data$Age *data$log_Total_bilirubin**

**data$age_heartrate = data$Age*data$Heart_rate**

**data$age_bapwv = data$Age*data$log_baPWV**

**data$age_abi = data$Age*data$ABI**

**data$age_sbp = data$Age*data$SBP**

**data$age_sbp2 = data$Age*data$SBP_sq**

**data$age_dbp = data$Age*data$DBP**

**data$age_dbp2 = data$Age*data$DBP_sq**

**data$SBP3 = data$SBP^3**

**data$ABI_cate = 2**

**data$ABI_cate[which(data$ABI<0.9)] = 1**

**data$ABI_cate[which(data$ABI>1.4)] = 3**

**data$ABI_cate = as.factor(data$ABI_cate)**

**#model 2 predictor selection:**

**my.variable.list1 = colnames(data)[c(3:4,12:36,38:72)]**

**res_m_diabdrug0 = data.frame(var=rep(NA,N),coe=rep(NA,N),P = rep(NA,N),sig = rep(0,N))**

**for(i in 1:N){**

**onel_m = paste("Surv(time, status) ~ Age+",my.variable.list1[i])**

**one.cox <- coxph(as.formula(onel_m), data = train_diabdrug0)**

**one_s=summary(one.cox)**

**res_m_diabdrug0[i,1] = my.variable.list1[i]**

**res_m_diabdrug0[i,2] = one_s$coefficients[2,1]**

**res_m_diabdrug0[i,3] = one_s$coefficients[2,5]**

**if(res_m_diabdrug0[i,3] < 0.05){**

**res_m_diabdrug0[i,4] = 1**

**}**

**}**

**#**

**print(res_m_diabdrug0[which(res_m_diabdrug0$sig==1),])**

**# age, smoking status, log-transformed total bilirubin, ABI, SBP, and the interaction of age and SBP were significant.**

**######## model 1 & 2:**

**model1 = as.formula(paste("Surv(time, status) ~ Age + Smoking_status + Total_cholesterol + log_HDL + SBP + drug_hypertension"))**

**sm1 = coxph(as.formula(model1), data = data)**

**model2 = as.formula(paste("Surv(time, status) ~ Age + Smoking_status + log_Total_bilirubin + SBP + age_sbp +ABI"))**

**sm2 = coxph(as.formula(model2), data = data)**

**#AIC**

**boot_aic1 <- function(sample, indices) {**

**sample_in <- sample[indices,]**

**sm1 = coxph(as.formula(model1), data = sample_in)**

**res = AIC(sm1)**

**return(res)**

**}**

**results_aic1 <- boot(data=data, statistic=boot_aic1, R=1000)**

**boot_aic2 <- function(sample, indices) {**

**sample_in <- sample[indices,]**

**sm2 = coxph(as.formula(model2), data = sample_in)**

**res = AIC(sm2)**

**return(res)**

**}**

**results_aic2 <- boot(data=data, statistic=boot_aic2, R=1000)**

**#BIC**

**boot_bic1 <- function(sample, indices) {**

**sample_in <- sample[indices,]**

**sm1 = coxph(as.formula(model1), data = sample_in)**

**res = BIC(sm1)**

**return(res)**

**}**

**results_bic1 <- boot(data=data, statistic=boot_bic1, R=1000)**

**boot_bic2 <- function(sample, indices) {**

**sample_in <- sample[indices,]**

**sm2 = coxph(as.formula(model2), data = sample_in)**

**res = BIC(sm2)**

**return(res)**

**}**

**results_bic2 <- boot(data=data, statistic=boot_bic2, R=1000)**

**################################**

**######model 3 LASSO**

**set.seed(1234)**

**library(glmnet)**

**x <- data.matrix(data[,c(3:4,12:36,38:72)])**

**y <- data.matrix(data[,c(which(colnames(data)=="time"),which(colnames(data)=="status"))])**

**lasso_fit_cv <- cv.glmnet(x,y,alpha=1,family = 'cox',standardize = TRUE)**

**lambda_use <- lasso_fit_cv$lambda.min**

**lasso_fit <- glmnet(x,y,lambda=lambda_use,alpha = 1,family = "cox",standardize = TRUE)**

**sm3 = survfit(lasso_fit, s = lambda_use, x = x, y = Surv(data$time, data$status))**

**#sm3$cumhaz[which(sm3$time==365*4)]**

**temp=rownames(lasso_fit$beta)[which(lasso_fit$beta!=0)]**

**model3 = as.formula(paste("Surv(time, status) ~",paste(temp[-length(temp)],collapse = "+")))**

**sm3 = coxph(as.formula(model3), data = data)**

**boot_aic3 <- function(sample, indices) {**

**sample_in <- sample[indices,]**

**sm3 = coxph(as.formula(model3), data = sample_in)**

**res = AIC(sm3)**

**return(res)**

**}**

**results_aic3 <- boot(data=data, statistic=boot_aic3, R=1000)**

**boot_bic3 <- function(sample, indices) {**

**sample_in <- sample[indices,]**

**sm3 = coxph(as.formula(model3), data = sample_in)**

**res = BIC(sm3)**

**return(res)**

**}**

**results_bic3 <- boot(data=data, statistic=boot_bic3, R=1000)**

**##########################**

**data$status4years = 0**

**data$status4years[which(data$status==1 & data$time <= 365*4)] = 1**

**#4-year incidence rate, model1：**

**CH=basehaz(sm1,centered =F)**

**baseline_surF = exp(-CH[which(CH$time==365*4),1])**

**fit = predict(sm1,data,type = "lp",se.fit=F,reference="zero")**

**risk1= 1 - baseline_surF^exp(fit)**

**##**

**CH=basehaz(sm1,centered =T)**

**baseline_surF_c = exp(-CH[which(CH$time==365*4),1])**

**zcoef0 <- ifelse(is.na(coef(sm1)), 0, coef(sm1))**

**offset0 <- sum(sm1$means * zcoef0)**

**risk1_c = 1 - baseline_surF_c^exp(fit - offset0)**

**x = paste(round(sm1$coefficients,3),"*",names(sm1$coefficients),collapse = " + ",sep = "")**

**form1 = paste("1 - ",round(baseline_surF_c,3),"^exp(",x," -",round(offset0,3),")",sep="")**

**results_cali_model1 = data.frame(auc=rep(NA,100),brier = rep(NA,100),hl = rep(NA,100),cindex=rep(NA,100))**

**boot_cali1 <- function(sample, indices) {**

**sample_in <- sample[indices,]**

**fit = predict(sm1,sample_in,type = "lp",se.fit=F,reference="zero")**

**risks_4years=1 - baseline_surF^exp(fit)**

**res1=roc(sample_in$status, risks_4years)$auc**

**#res2=brier(sample_in$status, risks_4years)**

**#res3=hoslem.test(sample_in$status, risks_4years,g=10)$p.value**

**#res4 = cindex(model1, sample_in)$cindex**

**#res = list(res1,res2,res3,res4)**

**return(res1)**

**}**

**boot_cali2 <- function(sample, indices) {**

**sample_in <- sample[indices,]**

**fit = predict(sm1,sample_in,type = "lp",se.fit=F,reference="zero")**

**risks_4years=1 - baseline_surF^exp(fit)**

**#res1=roc(sample_in$status, risks_4years)$auc**

**res2=brier(sample_in$status, risks_4years)**

**#res3=hoslem.test(sample_in$status, risks_4years,g=10)$p.value**

**#res4 = cindex(model1, sample_in)$cindex**

**#res = list(res1,res2,res3,res4)**

**return(res2)**

**}**

**boot_cali3 <- function(sample, indices) {**

**sample_in <- sample[indices,]**

**fit = predict(sm1,sample_in,type = "lp",se.fit=F,reference="zero")**

**risks_4years=1 - baseline_surF^exp(fit)**

**#res1=roc(sample_in$status, risks_4years)$auc**

**#res2=brier(sample_in$status, risks_4years)**

**res3=hoslem.test(sample_in$status, risks_4years,g=10)$p.value**

**#res4 = cindex(model1, sample_in)$cindex**

**#res = list(res1,res2,res3,res4)**

**return(res3)**

**}**

**boot_cali4 <- function(sample, indices) {**

**sample_in <- sample[indices,]**

**fit = predict(sm1,sample_in,type = "lp",se.fit=F,reference="zero")**

**risks_4years=1 - baseline_surF^exp(fit)**

**#res1=roc(sample_in$status, risks_4years)$auc**

**#res2=brier(sample_in$status, risks_4years)**

**#res3=hoslem.test(sample_in$status, risks_4years,g=10)$p.value**

**res4 = cindex(model1, sample_in)$cindex**

**#res = list(res1,res2,res3,res4)**

**return(res4)**

**}**

**results_cali_model1$auc <- boot(data=data, statistic=boot_cali1, R=1000)$t**

**results_cali_model1$brier <- boot(data=data, statistic=boot_cali2, R=1000)$t**

**results_cali_model1$hl <- boot(data=data, statistic=boot_cali3, R=1000)$t**

**results_cali_model1$cindex <- boot(data=data, statistic=boot_cali4, R=1000)$t**

**########**

**#4-year incidence rate, model2：**

**CH=basehaz(sm2,centered =F)**

**baseline_surF = exp(-CH[which(CH$time==365*4),1])**

**fit = predict(sm2,data,type = "lp",se.fit=F,reference="zero")**

**risk1= 1 - baseline_surF^exp(fit)**

**##**

**CH=basehaz(sm2,centered =T)**

**baseline_surF_c = exp(-CH[which(CH$time==365*4),1])**

**zcoef0 <- ifelse(is.na(coef(sm2)), 0, coef(sm2))**

**offset0 <- sum(sm2$means * zcoef0)**

**risk1_c = 1 - baseline_surF_c^exp(fit - offset0)**

**x = paste(round(sm2$coefficients,3),"*",names(sm2$coefficients),collapse = " + ",sep = "")**

**form2 = paste("1 - ",round(baseline_surF_c,3),"^exp(",x," -",round(offset0,3),")",sep="")**

**results_cali_model2 = data.frame(auc=rep(NA,100),brier = rep(NA,100),hl = rep(NA,100),cindex=rep(NA,100))**

**boot_cali1 <- function(sample, indices) {**

**sample_in <- sample[indices,]**

**fit = predict(sm2,sample_in,type = "lp",se.fit=F,reference="zero")**

**risks_4years=1 - baseline_surF^exp(fit)**

**res1=roc(sample_in$status, risks_4years)$auc**

**#res2=brier(sample_in$status, risks_4years)**

**#res3=hoslem.test(sample_in$status, risks_4years,g=10)$p.value**

**#res4 = cindex(model1, sample_in)$cindex**

**#res = list(res1,res2,res3,res4)**

**return(res1)**

**}**

**boot_cali2 <- function(sample, indices) {**

**sample_in <- sample[indices,]**

**fit = predict(sm2,sample_in,type = "lp",se.fit=F,reference="zero")**

**risks_4years=1 - baseline_surF^exp(fit)**

**#res1=roc(sample_in$status, risks_4years)$auc**

**res2=brier(sample_in$status, risks_4years)**

**#res3=hoslem.test(sample_in$status, risks_4years,g=10)$p.value**

**#res4 = cindex(model1, sample_in)$cindex**

**#res = list(res1,res2,res3,res4)**

**return(res2)**

**}**

**boot_cali3 <- function(sample, indices) {**

**sample_in <- sample[indices,]**

**fit = predict(sm2,sample_in,type = "lp",se.fit=F,reference="zero")**

**risks_4years=1 - baseline_surF^exp(fit)**

**#res1=roc(sample_in$status, risks_4years)$auc**

**#res2=brier(sample_in$status, risks_4years)**

**res3=hoslem.test(sample_in$status, risks_4years,g=10)$p.value**

**#res4 = cindex(model1, sample_in)$cindex**

**#res = list(res1,res2,res3,res4)**

**return(res3)**

**}**

**boot_cali4 <- function(sample, indices) {**

**sample_in <- sample[indices,]**

**fit = predict(sm2,sample_in,type = "lp",se.fit=F,reference="zero")**

**risks_4years=1 - baseline_surF^exp(fit)**

**#res1=roc(sample_in$status, risks_4years)$auc**

**#res2=brier(sample_in$status, risks_4years)**

**#res3=hoslem.test(sample_in$status, risks_4years,g=10)$p.value**

**res4 = cindex(model1, sample_in)$cindex**

**#res = list(res1,res2,res3,res4)**

**return(res4)**

**}**

**results_cali_model2$auc <- boot(data=data, statistic=boot_cali1, R=1000)$t**

**results_cali_model2$brier <- boot(data=data, statistic=boot_cali2, R=1000)$t**

**results_cali_model2$hl <- boot(data=data, statistic=boot_cali3, R=1000)$t**

**results_cali_model2$cindex <- boot(data=data, statistic=boot_cali4, R=1000)$t**

**########**

**########**

**#4-year incidence rate, model3 ：**

**CH=basehaz(sm3,centered =F)**

**baseline_surF = exp(-CH[which(CH$time==365*4),1])**

**fit = predict(sm3,data,type = "lp",se.fit=F,reference="zero")**

**risk1= 1 - baseline_surF^exp(fit)**

**##**

**CH=basehaz(sm3,centered =T)**

**baseline_surF_c = exp(-CH[which(CH$time==365*4),1])**

**zcoef0 <- ifelse(is.na(coef(sm3)), 0, coef(sm3))**

**offset0 <- sum(sm3$means * zcoef0)**

**risk1_c = 1 - baseline_surF_c^exp(fit - offset0)**

**x = paste(round(sm3$coefficients,3),"*",names(sm3$coefficients),collapse = " + ",sep = "")**

**form3 = paste("1 - ",round(baseline_surF_c,3),"^exp(",x," -",round(offset0,3),")",sep="")**

**results_cali_model3 = data.frame(auc=rep(NA,100),brier = rep(NA,100),hl = rep(NA,100),cindex=rep(NA,100))**

**boot_cali1 <- function(sample, indices) {**

**sample_in <- sample[indices,]**

**fit = predict(sm3,sample_in,type = "lp",se.fit=F,reference="zero")**

**risks_4years=1 - baseline_surF^exp(fit)**

**res1=roc(sample_in$status, risks_4years)$auc**

**#res2=brier(sample_in$status, risks_4years)**

**#res3=hoslem.test(sample_in$status, risks_4years,g=10)$p.value**

**#res4 = cindex(model1, sample_in)$cindex**

**#res = list(res1,res2,res3,res4)**

**return(res1)**

**}**

**boot_cali2 <- function(sample, indices) {**

**sample_in <- sample[indices,]**

**fit = predict(sm3,sample_in,type = "lp",se.fit=F,reference="zero")**

**risks_4years=1 - baseline_surF^exp(fit)**

**#res1=roc(sample_in$status, risks_4years)$auc**

**res2=brier(sample_in$status, risks_4years)**

**#res3=hoslem.test(sample_in$status, risks_4years,g=10)$p.value**

**#res4 = cindex(model1, sample_in)$cindex**

**#res = list(res1,res2,res3,res4)**

**return(res2)**

**}**

**boot_cali3 <- function(sample, indices) {**

**sample_in <- sample[indices,]**

**fit = predict(sm3,sample_in,type = "lp",se.fit=F,reference="zero")**

**risks_4years=1 - baseline_surF^exp(fit)**

**#res1=roc(sample_in$status, risks_4years)$auc**

**#res2=brier(sample_in$status, risks_4years)**

**res3=hoslem.test(sample_in$status, risks_4years,g=10)$p.value**

**#res4 = cindex(model1, sample_in)$cindex**

**#res = list(res1,res2,res3,res4)**

**return(res3)**

**}**

**boot_cali4 <- function(sample, indices) {**

**sample_in <- sample[indices,]**

**fit = predict(sm3,sample_in,type = "lp",se.fit=F,reference="zero")**

**risks_4years=1 - baseline_surF^exp(fit)**

**#res1=roc(sample_in$status, risks_4years)$auc**

**#res2=brier(sample_in$status, risks_4years)**

**#res3=hoslem.test(sample_in$status, risks_4years,g=10)$p.value**

**res4 = cindex(model1, sample_in)$cindex**

**#res = list(res1,res2,res3,res4)**

**return(res4)**

**}**

**results_cali_model3$auc <- boot(data=data, statistic=boot_cali1, R=1000)$t**

**results_cali_model3$brier <- boot(data=data, statistic=boot_cali2, R=1000)$t**

**results_cali_model3$hl <- boot(data=data, statistic=boot_cali3, R=1000)$t**

**set.seed(12011)**

**results_cali_model3$cindex <- boot(data=data, statistic=boot_cali4, R=1000)$t**

**sumst <- function(x){**

**m = mean(x)**

**up = quantile(x,0.95)**

**lo = quantile(x,0.05)**

**res = c(m,up,lo)**

**return(res)**

**}**

**m1=apply(results_cali_model1,2,sumst)**

**m2=apply(results_cali_model2,2,sumst)**

**m3=apply(results_cali_model3,2,sumst)**

**t1=apply(cbind(results_aic1$t,results_aic2$t,results_aic3$t),2,sumst)**

**t2=apply(cbind(results_bic1$t,results_bic2$t,results_bic3$t),2,sumst)**

**pst <- function(x){**

**paste(round(x[1],2)," (",round(x[3],2),", ",round(x[2],2),")",sep="")**

**}**

**pst3 <- function(x){**

**paste(round(x[1],3)," (",round(x[3],3),", ",round(x[2],3),")",sep="")**

**}**

**apply(t1,2,pst3)**

**apply(t2,2,pst3)**

**apply(m1,2,pst3)**

**apply(m2,2,pst3)**

**apply(m3,2,pst3)**

**Supplementary Table 1.** The results of age-adjusted univariable statistics.

| Variable | Coefficient | Standard error | P-value |
| --- | --- | --- | --- |
| hyperlipidemia drug usage | 0.36 | 0.39 | 0.357 |
| hypertension drug usage | 0.06 | 0.20 | 0.785 |
| Diabetes drug usage | 0.32 | 0.31 | 0.294 |
| Drinking | -0.87 | 0.40 | 0.028 |
| Smoking status | 0.39 | 0.21 | 0.059 |
| Urine protein | 0.37 | 0.35 | 0.282 |
| Hypertension | 0.01 | 0.19 | 0.950 |
| Diabetes | 0.22 | 0.25 | 0.360 |
| Hyperlipidemia | 0.13 | 0.21 | 0.534 |
| BMI | -0.05 | 0.03 | 0.109 |
| Average PLT volume | -0.08 | 0.05 | 0.122 |
| log(RBC distribution width) | -0.56 | 1.45 | 0.697 |
| log(SII) | 0.13 | 0.18 | 0.465 |
| log(Fasting blood glucose) | 0.50 | 0.48 | 0.296 |
| Hemoglobin A1c | 0.01 | 0.01 | 0.436 |
| log(HDL) | -0.38 | 0.40 | 0.337 |
| LDL | -0.13 | 0.12 | 0.295 |
| Total cholesterol | -0.04 | 0.10 | 0.673 |
| log(Triglyceride) | 0.04 | 0.16 | 0.793 |
| Serum uric acid | <0.01 | <0.01 | 0.840 |
| eGFR | <0.01 | 0.01 | 0.502 |
| log(Total bilirubin) | -0.66 | 0.25 | 0.008 |
| Heart rate | <0.01 | 0.01 | 0.926 |
| log(baPWV) | 0.13 | 0.51 | 0.803 |
| ABI | -2.33 | 1.08 | 0.031 |
| SBP | <0.01 | 0.01 | 0.665 |
| DBP | <0.01 | 0.01 | 0.562 |
| log(baPWV)^2 | 0.01 | 0.03 | 0.848 |
| BMI^2 | <0.01 | <0.01 | 0.101 |
| SBP^2 | <0.01 | <0.01 | 0.662 |
| DBP^2 | <0.01 | <0.01 | 0.649 |
| Hemoglobin_A1c^2 | <0.01 | <0.01 | 0.168 |
| Age*drinking | -0.02 | 0.01 | 0.025 |
| Age *smoking | 0.01 | <0.01 | 0.073 |
| Age *Urine_protein | 0.01 | 0.01 | 0.284 |
| Age *hypertension | <0.01 | <0.01 | 0.966 |
| Age *diabetes | <0.01 | <0.01 | 0.361 |
| Age *hyperlipidemia | <0.01 | <0.01 | 0.628 |
| Age *BMI | <0.01 | <0.01 | 0.104 |
| Age *BMI^2 | <0.01 | <0.01 | 0.094 |
| Age *averagePLT | <0.01 | <0.01 | 0.124 |
| Age *RBC | -0.01 | 0.02 | 0.697 |
| Age *SII | 0.00 | 0.00 | 0.525 |
| Age *fasting_glu | 0.01 | 0.01 | 0.461 |
| Age *Hemoglobin_A1c | 0.00 | 0.00 | 0.605 |
| Age *Hemoglobin_A1c^2 | 0.00 | 0.00 | 0.281 |
| Age *HDL | -0.01 | 0.01 | 0.360 |
| Age *LDL | 0.00 | 0.00 | 0.227 |
| Age *Tcholesterol | 0.00 | 0.00 | 0.661 |
| Age *Triglyceride | 0.00 | 0.00 | 0.820 |
| Age *serum_uric_acid | 0.00 | 0.00 | 0.789 |
| Age *eGFR | 0.00 | 0.00 | 0.606 |
| Age *Tbilirubin | -0.01 | 0.00 | 0.011 |
| Age *heart_rate | 0.00 | 0.00 | 0.962 |
| Age *baPWV | 0.00 | 0.01 | 0.899 |
| Age *ABI | -0.04 | 0.02 | 0.025 |
| Age *SBP | 0.00 | 0.00 | 0.464 |
| Age *SBP^2 | 0.00 | 0.00 | 0.461 |
| Age *DBP | 0.00 | 0.00 | 0.653 |
| Age *DBP^2 | 0.00 | 0.00 | 0.748 |
| SBP^3 | 0.00 | 0.00 | 0.673 |

TRIPOD Checklist: Prediction Model Development and Validation

| **Section/Topic** | **Item** |  | **Checklist Item** | **Page** |
| --- | --- | --- | --- | --- |
| **Title and abstract** | | | | |
| Title | 1 | D;V | Identify the study as developing and/or validating a multivariable prediction model, the target population, and the outcome to be predicted. | 1 |
| Abstract | 2 | D;V | Provide a summary of objectives, study design, setting, participants, sample size, predictors, outcome, statistical analysis, results, and conclusions. | 3 |
| **Introduction** | | | | |
| Background and objectives | 3a | D;V | Explain the medical context (including whether diagnostic or prognostic) and rationale for developing or validating the multivariable prediction model, including references to existing models. | 5 |
|  | 3b | D;V | Specify the objectives, including whether the study describes the development or validation of the model or both. | 6 |
| **Methods** | | | | |
| Source of data | 4a | D;V | Describe the study design or source of data (e.g., randomized trial, cohort, or registry data), separately for the development and validation data sets, if applicable. | 6-7 |
|  | 4b | D;V | Specify the key study dates, including start of accrual; end of accrual; and, if applicable, end of follow-up. | 7 |
| Participants | 5a | D;V | Specify key elements of the study setting (e.g., primary care, secondary care, general population) including number and location of centres. | 7 |
|  | 5b | D;V | Describe eligibility criteria for participants. | 7 |
|  | 5c | D;V | Give details of treatments received, if relevant. | NA |
| Outcome | 6a | D;V | Clearly define the outcome that is predicted by the prediction model, including how and when assessed. | 8 |
|  | 6b | D;V | Report any actions to blind assessment of the outcome to be predicted. | NA |
| Predictors | 7a | D;V | Clearly define all predictors used in developing or validating the multivariable prediction model, including how and when they were measured. | 6-7 |
|  | 7b | D;V | Report any actions to blind assessment of predictors for the outcome and other predictors. | NA |
| Sample size | 8 | D;V | Explain how the study size was arrived at. | 9 |
| Missing data | 9 | D;V | Describe how missing data were handled (e.g., complete-case analysis, single imputation, multiple imputation) with details of any imputation method. | 10 |
| Statistical analysis methods | 10a | D | Describe how predictors were handled in the analyses. | 8 |
|  | 10b | D | Specify type of model, all model-building procedures (including any predictor selection), and method for internal validation. | 10-11 |
|  | 10c | V | For validation, describe how the predictions were calculated. | 11-12 |
|  | 10d | D;V | Specify all measures used to assess model performance and, if relevant, to compare multiple models. | 11-12 |
|  | 10e | V | Describe any model updating (e.g., recalibration) arising from the validation, if done. | NA |
| Risk groups | 11 | D;V | Provide details on how risk groups were created, if done. | 11 |
| Development vs. validation | 12 | V | For validation, identify any differences from the development data in setting, eligibility criteria, outcome, and predictors. | NA |
| **Results** | | | | |
| Participants | 13a | D;V | Describe the flow of participants through the study, including the number of participants with and without the outcome and, if applicable, a summary of the follow-up time. A diagram may be helpful. | 13 |
|  | 13b | D;V | Describe the characteristics of the participants (basic demographics, clinical features, available predictors), including the number of participants with missing data for predictors and outcome. | 13 |
|  | 13c | V | For validation, show a comparison with the development data of the distribution of important variables (demographics, predictors and outcome). | 14,32 |
| Model development | 14a | D | Specify the number of participants and outcome events in each analysis. | 13,29 |
|  | 14b | D | If done, report the unadjusted association between each candidate predictor and outcome. | NA |
| Model specification | 15a | D | Present the full prediction model to allow predictions for individuals (i.e., all regression coefficients, and model intercept or baseline survival at a given time point). | 14-15 |
|  | 15b | D | Explain how to the use the prediction model. | 16 |
| Model performance | 16 | D;V | Report performance measures (with CIs) for the prediction model. | 14,15,32,34 |
| Model-updating | 17 | V | If done, report the results from any model updating (i.e., model specification, model performance). | NA |
| **Discussion** | | | | |
| Limitations | 18 | D;V | Discuss any limitations of the study (such as nonrepresentative sample, few events per predictor, missing data). | 18-20 |
| Interpretation | 19a | V | For validation, discuss the results with reference to performance in the development data, and any other validation data. | 20 |
|  | 19b | D;V | Give an overall interpretation of the results, considering objectives, limitations, results from similar studies, and other relevant evidence. | 20-21 |
| Implications | 20 | D;V | Discuss the potential clinical use of the model and implications for future research. | 21-22 |
| **Other information** | | | | |
| Supplementary information | 21 | D;V | Provide information about the availability of supplementary resources, such as study protocol, Web calculator, and data sets. | 17,18,21 |
| Funding | 22 | D;V | Give the source of funding and the role of the funders for the present study. | 24 |

*Items relevant only to the development of a prediction model are denoted by D, items relating solely to a validation of a prediction model are denoted by V, and items relating to both are denoted D;V. We recommend using the TRIPOD Checklist in conjunction with the TRIPOD Explanation and Elaboration document.
